# Hairy Cell Leukemia: Hepatic Affinity Status Post Splenectomy

**DOI:** 10.7759/cureus.39830

**Published:** 2023-06-01

**Authors:** Gurneel K Dhanesar, Jonathan Livingston, Michael Maroules, Sheue H Lee

**Affiliations:** 1 Internal Medicine, New York Medical College at St. Mary’s General Hospital, Passaic and St. Clare’s Health, Denville, USA; 2 Hematology and Oncology, St. Mary's General Hospital, Passaic, USA

**Keywords:** rituximab, vemurafenib, cytopenia, hepatology, hematology-oncology, bcell, splenectomy, hairy cell leukemia, lymphoma, oncology

## Abstract

Hairy cell leukemia (HCL) is a rare neoplasm of the B-cell lineage that is characterized by an indolent course and infiltration of the spleen, the bone marrow, and the reticuloendothelial system. Splenectomy is considered an effective treatment for peripheral cytopenia in patients with HCL. Hepatic involvement of hairy cells with infiltration of the sinusoidal endothelial cells is rarely reported in the literature and is not well understood. We present the case of an 88-year-old male with a history of traumatic splenectomy who was found to have a relapse of classic hairy cell leukemia within the hepatic portal system.

## Introduction

Hairy cell leukemia (HCL) is a hematopoietic and lymphoid malignancy that is a distinct entity classified by the World Health Organization. It is a rare cancer that represents approximately 2% of all leukemias and 1% of lymphoid neoplasms. The median age of onset is 50 to 55 years, and there is a male predilection with a male-to-female ratio of four-to-one [1]. HCL is a mature B-cell lymphoma characterized histologically as mononuclear cells with ovoid nuclei and abundant variable 'hair-like' cytoplasmic projections that circulate in the peripheral blood [2]. These hairy cells have a specific immunophenotype and generally express clonal clusters of differentiation (CD) antigen markers CD19, CD20, and CD22 that can be identified by flow cytometry [3]. No formal genetic abnormalities have been included in the diagnostic criteria; however, BRAF600E mutations have been implicated in the vast majority of HCL cases. The circulating neoplastic cells infiltrate the splenic red pulp and bone marrow and can lead to pancytopenia and subsequent splenomegaly [1]. The disease process is typically indolent and asymptomatic, but patients can present clinically with generalized weakness, fatigue, hemorrhagic abnormalities, and infections. Organ involvement commonly includes infiltration of the spleen and has only been rarely reported to involve the liver and lymph nodes. In this case, we present an 88-year-old male who was diagnosed with hairy cell leukemia involving the hepatic portal system and peri-portal vasculature status post-splenectomy.

## Case presentation

**Figure 1:**
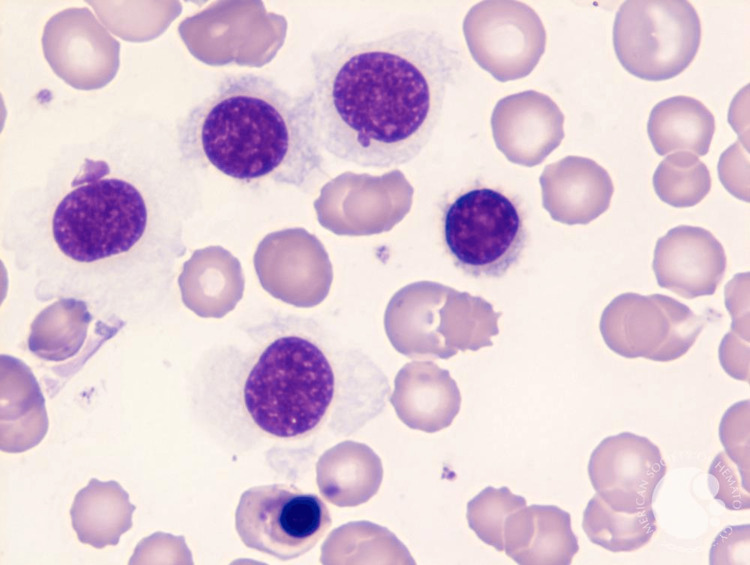
Peripheral blood smear Revealing classic hairy cell morphology, including mononuclear cells with large ovoid nuclei and abundantly variably organized cytoplasmic projections. (This image was originally published in the American Society of Hematology Image Bank and is from a separate reported case of HCL). HCL: hairy cell luekemia

**Figure 2:**
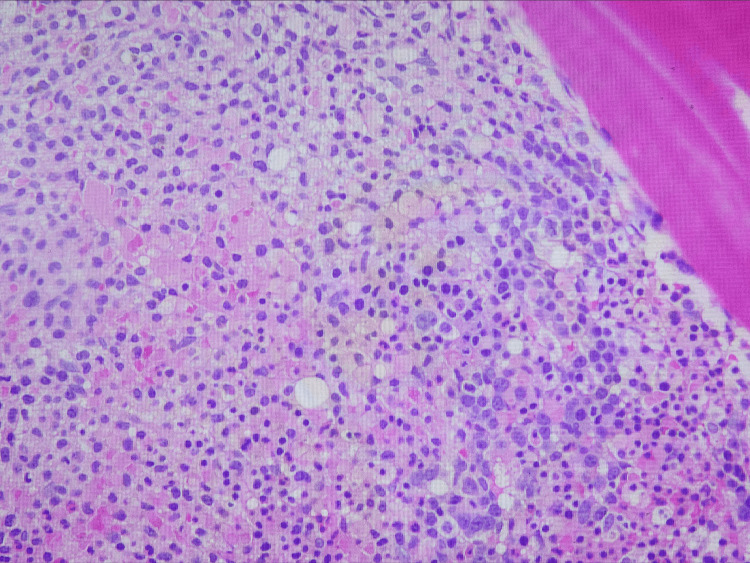
Bone core biopsy; hematoxylin & eosin stain Bone marrow revealing a relatively inconspicuous interstitial infiltrate of medium-sized, relatively monotonous lymphoid cells with ample cytoplasm and distinct cytoplasmic borders.

**Figure 3:**
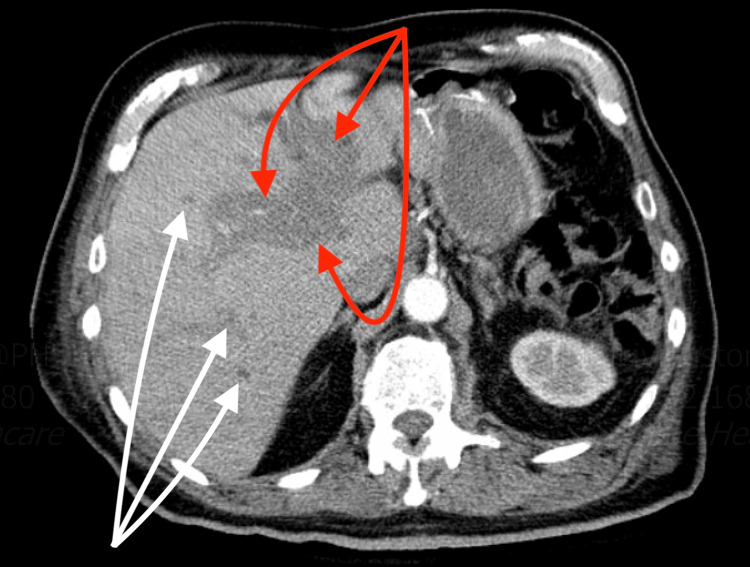
Non-contrast CT scan of the abdomen and pelvis Tumor infiltration (red arrows) of the hepatic peri-portal system of the liver (white arrows). CT: computed tomography

**Figure 4:**
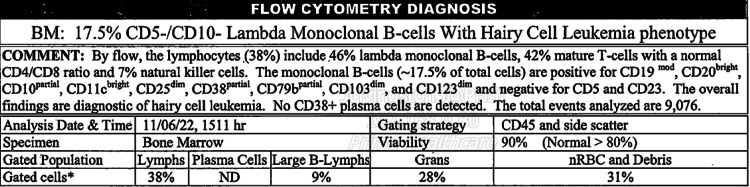
Flow cytometry diagnosis summary

**Figure 5:**
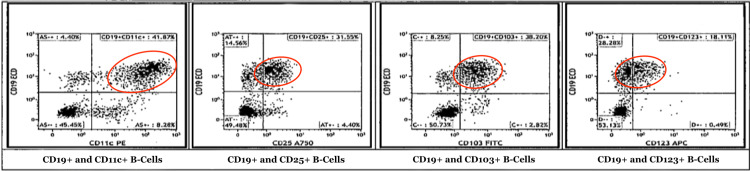
Flow cytometry analysis Flow cytometry analysis revealing clonal cluster of differentiation (CD) 19 positive B-cells with CD11c+, CD25+, CD103+, and CD123+ markers which are further delineated within the red ovals. These findings are highly suggestive of HCL. CD: cluster of differentiation; HCL: hairy cell leukemia

An 88-year-old male presented to the hospital complaining of generalized fatigue and dyspnea for several weeks. His past medical history was significant for prostate cancer, undergoing treatment with leuprolide and nilutamide; gastrointestinal stromal tumor of the stomach, status post-resection in 2017; and hairy cell leukemia with infiltration of the bone marrow that was diagnosed in 2018 and managed with pentostatin and rituximab. The patient also underwent a splenectomy following a traumatic splenic rupture during a motor vehicle accident in 2016. On physical examination, the patient was afebrile, normotensive, and tachycardic, with a heart rate in the 130s. There was no lymphadenopathy, and the abdomen was soft, non-tender, and non-distended without hepatomegaly. Diagnostic workup revealed a low-to-normal leukocyte count of 3.7 x 103/uL (reference range: 3.5-10.5 x 103 uL), normocytic anemia with a hemoglobin of 6.6 g/dL (reference range: 13.5-17.5 g/dL), platelet count of 40 x 103/uL (reference range: 150-450 x 103uL), alanine aminotransferase 147 U/L (reference range: 9-46 U/L), aspartate aminotransferase 56 U/L (reference range: 10-36 U/L) and a total bilirubin of 1.4 mg/dL (reference range: 0.2-1.2 mg/dL). The peripheral blood smear and bone core biopsy showed classic morphologic findings of HCL, as shown and outlined in Figure 1 and Figure 2, respectively. An abdominal ultrasound showed findings consistent with a necrotic mass in the left hepatic lobe, and a subsequent computed tomography (CT) scan of the abdomen revealed a tumor in the hepatic portal and peri-portal regions with bile duct obstruction, as seen in Figure 3. A CT-guided biopsy of the liver mass was performed and supported a diagnosis of HCL with BRAF V600E mutation. Detailed flow cytometry analyses are summarized and depicted in Figure 4 and Figure 5 respectively.

The patient was started on a course of vemurafenib and rituximab in consideration of HCL relapse and discharged home. Shortly after treatment initiation, the patient was readmitted for severe weakness and hematuria. Laboratory workup at that time was significant for pancytopenia with a hemoglobin of 7.5 g/dL (reference range: 13.5-17.5), leukocyte count of 4.5 x 10^3 µL (reference range: 3.5-10.5 × 103 µL), and a platelet count of 77 x 10^3 µL (reference range: 150-450 × 103 µL). The patient ultimately decompensated, experienced a pulseless cardiopulmonary arrest, and passed away despite resuscitative efforts. Arterial blood gas drawn during the time of arrest showed a significant metabolic acidosis with a pH of 6.682 (reference range: 7.340- 7.440) with a contributing lactate of 22.7 mmol/L (reference range: 0-2 mmol/L).

## Discussion

Hairy cell leukemia is a lymphoproliferative disorder of mature B-cells that typically circulate through and proliferate in the reticuloendothelial system, leading to bone marrow suppression and splenomegaly [3,4]. Approximately 25% of cases are found incidentally in asymptomatic patients, with the remaining cases presenting symptoms that are a consequence of splenomegaly and cytopenias, including but not limited to generalized weakness, fatigue, infections, and bleeding. Lymphadenopathy and hepatomegaly are rare features associated with HCL and are found in approximately 10% and 20% of cases, respectively [1]. It is important to note that hepatomegaly was not appreciated in our patient, who was found to have hepatic involvement of HCL.

Ideally, the diagnosis of HCL is made with a peripheral blood smear, flow cytometry, and trephine bone marrow biopsy and/or aspiration [5]. The peripheral blood smear will reveal distinct morphologic characteristics consisting of small-to-medium-sized lymphoid cells measuring approximately 10-20 millimeters in diameter. Additionally, the hairy cells contain eccentrically located nuclei with fine, spongy chromatin and have blue-gray cytoplasm with circumferential, shaggy, irregular projections [6]. The immunophenotype and genetic markers generally associated with HCL are identified via flow cytometry. These include but are not limited to CD11, CD25, CD103, CD123, BRAF, MAP2K1, and KLF2 mutations. These neoplastic B-lymphocytes are arrested at a late stage of differentiation, showing strong light-chain kappa (κ) or lambda (λ) restricted surface immunoglobulin [7]. The extent of bone marrow involvement is elucidated with a trephine aspirate and bone marrow biopsy; however, a dry tap from significant fibrosis and hypocellularity often confounds the aspiration and biopsy results. Furthermore, HCL cells are believed to up-regulate the expression of cytoskeleton components such as CD20, Annexin-A1, and BCL1 that can more accurately be assessed with immunohistochemical stains [7-8]. In our case, the patient had an initial diagnosis of HCL in 2018 that was treated. A subsequent investigation during the current hospitalization, including a CT-guided biopsy of the liver, was consistent with HCL relapse with hepatic involvement.

The pathogenesis of HCL is hypothesized to be caused by genetic mutations involved in cell survivability and is thought to be at least partially dependent on the local cellular microenvironment. The kinase-activator BRAF-V600E has been explicitly identified as a culprit gene mutation. This leads to activation of the rapidly accelerating fibrosarcoma-mitogen-activated protein kinase-extracellular signal-regulated kinase (RAF-MEK-ERK) pathway, which subsequently results in anti-apoptotic activity leading to increased cellular proliferation and ultimately malignancy. Another common mutation associated with HCL is the cyclin-dependent kinase inhibitor 1B/p27 (CKDN1B/p27 cell cycle inhibitor inactivation). CDKN1B encodes for the p27 protein, which is an important regulator of the cell cycle and plays a significant role in cellular activities such as inhibition of cyclin-dependent kinase, regulation of apoptosis, and interaction with the cytoskeleton [3,9-11]. Macroscopically, HCL-associated fibrosis occurs after infiltration into hyaluronic-rich tissues such as bone marrow and hepatic portal tracts. In the liver, neoplastic hairy cells exhibit angiomatous-like characteristics by infiltrating and proliferating in hepatic sinusoids. Traditionally, splenomegaly occurs from hyperplasia of the splenic red pulp and HCL infiltration [7, 12]. However, there are almost no cases in the current literature highlighting a reason for hepatic affinity and infiltration as was observed in this particular case.

The mainstay of therapy for HCL is purine analogue induction therapy with either cladribine or pentostatin [13]. In patients with symptomatic and significant splenomegaly measuring greater than 10 centimeters below the costal margin, a splenectomy may be considered [1]. A recent phase-two, single-center trial on 30 patients with uncontrolled HCL was performed that assessed the safety and efficacy of treatment with vemurafenib and rituximab and was found to be associated with a complete therapeutic response. Furthermore, in patients with HCL relapse, the disease was found to be progressively less sensitive to purine analogs and was associated with increased hematologic, toxic, and immunological side effects. A complete response was seen in 87% of patients, with minimal residual disease-negative status in 60% and relapse-free survival of 85% at a median of 34 months [13]. In our patient, we believe that hairy cell infiltration of the hepatic portal system occurred due to the history of splenectomy, which created a relatively more favorable microenvironment for disease progression and proliferation in the absence of splenic sequestration of neoplastic cells. His initial occurrence of HCL was managed with pentostatin and rituximab approximately four years before the re-occurrence, and non-myelotoxic therapy with vemurafenib and rituximab was considered for HCL relapse in the liver.

## Conclusions

Disorders causing morbidity in the form of B-symptoms are generally an adverse prognostic feature. The incidence and prognosis of hairy cell leukemia and relapse involving the liver in patients with splenectomy are not well reported in the literature, and it is imperative that further investigation be pursued to better understand the pathogenesis, therapeutic strategies, and prognosis of HCL and hepatic burden.
